# Advances in pathogenesis, novel therapeutic strategies and interventions for age-related hearing loss

**DOI:** 10.3389/fnmol.2026.1848164

**Published:** 2026-05-29

**Authors:** Zhiyang Wen, Yinfei Liang, Dongxue Wu, Huiqing Wu, Zhicheng Li, Gendi Yin, Xiangli Zeng

**Affiliations:** 1 Department of Otolaryngology-Head & Neck Surgery, The Third Affiliated Hospital of Sun Yat-sen University, Guangzhou, China; 2 Center of Dizziness and Tinnitus Diagnosis and Treatment, The Third Affiliated Hospital of Sun Yat-sen University, Guangzhou, China

**Keywords:** age-related hearing loss, gene therapy, inflammation, oxidative stress, presbycusis

## Abstract

Age-related hearing loss (ARHL), or presbycusis, represents one of the most prevalent sensory dysfunctions in the elderly people. It is characterized by progressive hearing deterioration that significantly impairs quality of life and increases the risk of comorbidities including cognitive impairment, depression, and social isolation. The pathogenesis of ARHL involves complex structural alterations such as degeneration of cochlear hair cells, loss of spiral ganglion neurons, and stria vascularis atrophy, coupled with molecular and cellular dysregulations including oxidative stress, inflammation, autophagy dysfunction, and cellular senescence. Meanwhile, genetic predisposition and environmental factors contribute to its development. Current conventional management primarily relies on hearing aids, which fail to reverse disease progression, while effective pharmacological interventions remain unavailable. Recent years have witnessed promising developments in innovative approaches such as gene therapy, stem cell-based regenerative medicine, nanotechnology-based delivery systems, and early biomarker screening, offering new directions for precise ARHL intervention. The purpose of this review is to provide a comprehensive overview of the current state of research in ARHL, with the goal of advancing our understanding, treatment and interventions of presbycusis.

## Introduction

Age-related hearing loss is a progressive auditory impairment primarily stemming from the degeneration of peripheral auditory systems (such as cochlear hair cells), declining central auditory processing capacity, and associated cognitive functional changes with advancing age (Huang et al., 2023; Tang et al., 2023). Its pathological mechanisms are complex, involving various biological processes including oxidative stress, inflammation, mitochondrial dysfunction, and autophagy abnormalities (He et al., 2021; Affortit et al., 2022; Schubert et al., 2024; Ding et al., 2025). Research also suggests that early-stage “hidden hearing loss” may be linked to age-related cochlear changes and could represent a prodromal phase preceding overt age-related hearing loss (Krist et al., 2021). From an epidemiological perspective, this condition exhibits extremely high prevalence rates that rise markedly with age. Among individuals over 60 years old, the prevalence reaches 65% (Ding et al., 2025). U.S. data indicate approximately 16% of adults report hearing difficulties, affecting about one-third of those aged 65 and older, with prevalence exceeding 90% in the over -80 population (Ahmed et al., 2025; Kolo et al., 2025; Nieman, 2025). Age-related hearing loss is not only a common sensory disorder but also poses significant health and societal burdens, ranking as the third leading cause of disability worldwide (Tang et al., 2023). It is closely associated with cognitive impairment, dementia, depression, increased fall risk, higher hospitalization rates, and social isolation (Brewster et al., 2021; Maidment et al., 2023; Kolo et al., 2025). Furthermore, its connection with poor cardiometabolic health suggests systemic factors may contribute to its development (Maidment et al., 2023). Risk factors for this condition encompass both genetic and environmental aspects. Genetic predisposition has been confirmed to contribute to hearing loss (Paciello et al., 2023a), while environmental noise exposure exhibits synergistic exacerbating effects with age-related hearing loss (Hu et al., 2023; Lee et al., 2025). Regarding lifestyle factors, oxidative stress and inflammation are considered potentially modifiable risk factors (Ding et al., 2025). In summary, age-related hearing loss is a multifactorial disease characterized by high prevalence and significant health impacts. Current interventions like hearing aids can alleviate symptoms but lack effective treatments capable of completely reversing or halting disease progression (Sun G. et al., 2025). Future research needs to elucidate its molecular mechanisms to facilitate the development of targeted therapeutic strategies.

Currently, the treatment of ARHL faces challenges, with no drugs or interventions capable of stopping its progression or restoring hearing (Cassinotti et al., 2022; Castelli et al., 2023; Kolo et al., 2025). The main research challenges include: increased genetic research difficulty due to pathological phenotypic heterogeneity (Ahmed et al., 2025); unclear mechanisms of hidden hearing loss hindering diagnostic and therapeutic progress (Schubert et al., 2024); insufficient exploration of the role of the central auditory system in dementia-related condition (Boons et al., 2025). Emerging intervention directions include: antioxidant strategies targeting oxidative stress (Lee et al., 2025); drug development based on autophagy regulation, such as DLK/JNK3 pathway (Schubert et al., 2024; Ding et al., 2025); using nanoparticle drug delivery technology to improve cochlear-targeted therapy (Schubert et al., 2024); and anti-aging therapies to clear senescent cells (Chen et al., 2025). From a public health perspective, ARHL has a certain preventability, and controlling lifestyle risk factors (such as reducing noise exposure and managing oxidative stress) may delay its onset (Tang et al., 2023; Reed et al., 2025). Early screening also holds significant value: pure-tone audiometry and distortion product otoacoustic emissions can serve as sensitive indicators of cognitive decline risk (Medel et al., 2024); polygenic risk scores may help in early risk assessment for ARHL (Wang et al., 2024). Therefore, the high prevalence of ARHL, its complex pathological mechanisms, strong association with major diseases such as dementia and depression, and the current lack of effective treatments highlight the urgency of addressing it as a global public health priority. In-depth research into its molecular mechanisms, exploration of targeted therapies such as anti-aging and autophagy regulation, and integrating hearing interventions with cognitive health management are key research directions for the future (Hu et al., 2023; Maidment et al., 2023; Paciello et al., 2023a; Wang et al., 2024; Sun G. et al., 2025). Cx26 ubiquitination mediated by cochlear ER stress is the main cause of Cx26 degradation in the cochlea of aged mice. Bai et al. analyzed Cx26 expression patterns in C57BL/6J mice across different age groups and confirmed an age-dependent downregulation of Cx26 in the mouse cochlea. They observed concurrent upregulation of endoplasmic reticulum (ER) stress markers Glucose-regulated protein 78 (GRP78) and protein disulfide isomerase (PDI) in aged cochlear tissues. Furthermore, experiments demonstrated that ER stress significantly reduced Cx26 protein levels in cochlear explants (Bai et al., 2025).

## Pathophysiological mechanisms of age-related hearing loss

### Overview of the pathophysiological mechanisms of age-related hearing loss

The pathogenesis of age-related hearing loss (ARHL) is multifactorial, involving a complex interplay of physiological and pathological mechanisms. In addition to aging, noise exposure, and ototoxic drugs, various age-related conditions such as hypertension, hyperglycemia, and hyperlipidemia may alter the osmotic pressure of the labyrinthine lymph fluid in the inner ear, thereby compromising cochlear structure and function. Other contributing factors, including ototoxicity, estrogen levels, and oxidative stress mediated by free radicals, have also been implicated (Lasisi et al., 2010; Yu et al., 2025). Increasing attention has been directed toward the role of genetic factors, particularly those involving mitochondrial genes, in the development of ARHL (Yang J. et al., 2023). The core structural basis stems from progressive damage to key components within the cochlea, including the loss of hair cells, degeneration of spiral ganglion neurons, and atrophy of the stria vascularis (Zhao X. et al., 2025). According to postmortem histopathological studies, a variety of pathological changes occur in the inner ear of patients with presbycusis, such as the atrophy of the stria vascularis (SV) and loss of fibrocytes of the spiral ligament (SL) in the lateral wall of the cochlea, decrease of sensory hair cells and degeneration of the auditory nerve (Ohlemiller, 2004; Bowl and Dawson, 2019). These directly lead to sensorineural hearing loss. The cochlear lateral wall degeneration is an important pathological change in aging. Potassium-secreting cells in the cochlea’s lateral wall often degenerate. The degeneration reduces the force that drives ions into the sensory cells during sound stimulation, which is traditionally thought to explain the loss of hearing. At the molecular and cellular level, oxidative stress is recognized as a core driver. The accumulation of reactive oxygen species (ROS) leads to cellular damage by damaging lipids and proteins, as well as attacking DNA directly or indirectly, leading to various pathologies, cancer, and aging, and leads to DNA damage and mitochondrial dysfunction in cochlear cells (Rivas-Chacon et al., 2021). ROS can also trigger abnormal autophagy via pathways such as DLK/JNK3, collectively accelerating cellular senescence and apoptosis (Suzuki et al., 2024; Ding et al., 2025). Cellular senescence itself and its associated senescence-associated secretory phenotype (SASP) release inflammatory factors, further exacerbating microenvironmental damage in cochlear tissues, making it a potential therapeutic target (Chen et al., 2025). Early pathological events often manifest as synaptic loss between inner hair cells and spiral ganglion neurons, which is considered the basis of “hidden hearing loss” (Cassinotti et al., 2022; Schubert et al., 2024). Additionally, a study demonstrates that the dysfunction of Kv7.4 potassium channels in outer hair cells disrupts potassium ion homeostasis. Impaired surface expression or reduced activity of the K_*V*_7.4 channel leads to functional impairment and has been associated with age-related hearing loss in human hereditary deafness DFNA2. The central role of the K_*V*_7.4 channel for OHC function and survival has been demonstrated by genetic ablation in *Kcnq4*^–/–^mice and loss-of-function mutations leading to progressive hearing loss and slow degeneration of OHCs (Schubert et al., 2024). The loss of the K_*V*_7.4 in OHCs can result in chronic depolarization, which can consequently lead to their degeneration due to chronic cellular stress (Ruttiger et al., 2004). It directly affects hair cell survival and function (Peixoto Pinheiro et al., 2022; Schubert et al., 2024).

Inflammation and immune responses run throughout the course of ARHL, interacting with oxidative stress to cause microvascular changes and inflammatory factor release, thereby exacerbating stria vascularis atrophy and hair cell damage (Tang et al., 2023; Clark et al., 2024). This process is often triggered and amplified by environmental risk factors such as noise or chemical exposure) (Tang et al., 2023). The impact of ARHL is not limited to the periphery; it also induces significant changes in central auditory processing, including abnormal alterations in brain neuroplasticity, which are closely associated with cognitive decline and increased depression risk, forming a vicious cycle (Brewster et al., 2021; Huang et al., 2023; Sharma et al., 2025).

Multiple modifiable lifestyle and environmental risk factors accelerate ARHL development through the aforementioned mechanisms, including noise exposure, ototoxic substances, smoking, and cardiovascular diseases, primarily by exacerbating oxidative stress and inflammation (Tang et al., 2023). Meanwhile, genetic and epigenetic factors constitute the intrinsic basis of disease heterogeneity. Genome-wide association studies have identified multiple relevant genes (e.g., TRα1), which increase susceptibility by influencing pathways such as oxidative stress and autophagy (Liu et al., 2021; Affortit et al., 2022). A recent study found that the p43^–/–^ mice exhibit no obvious hearing loss in juvenile stages, but that these mice developed a premature, and more severe, ARHL resulting from the loss of cochlear sensory outer and inner hair cells and degeneration of spiral ganglion neurons. Exacerbated ARHL in p43^–/–^ mice was associated with the early occurrence of a drastic fall of SIRT1 expression, together with an imbalance between pro-apoptotic Bax, p53 expression, and anti-apoptotic Bcl2 expression, as well as an increase in mitochondrial dysfunction, oxidative stress, and inflammatory process (Affortit et al., 2021) (Table 1).

**TABLE 1:** Pathophysiological mechanisms of age-related hearing loss.

Mechanism category	Core pathological changes	Primary effects
Auditory structural degeneration	IHC/OHC loss; SGN degeneration; Stria vascularis atrophy; Kv7.4 channel dysfunction; IHC-SGN synaptopathy	Loss of sound transduction; Impaired signal transmission; “Hidden hearing loss” (early stage)
Core molecular mechanisms	Oxidative stress (ROS); NLRP3 inflammasome activation; Cellular senescence (SASP); Dysregulated autophagy; Mitochondrial dysfunction	Cochlear cell damage; Chronic inflammation; Accelerated tissue aging
Genetic factors	Common GWAS variants; Rare Mendelian variants (GJB2, OPA1); Gene-environment interactions	Increased disease susceptibility; Heterogeneous clinical phenotypes
Environmental/lifestyle factors	Noise exposure; Ototoxic chemicals; Smoking; Metabolic disorders (diabetes/obesity)	Synergize with aging to exacerbate oxidative stress and inflammation
Central nervous system changes	Auditory cortex remodeling; Brain atrophy; White matter damage	Central auditory processing disorder; Linked to cognitive decline and depression

### Aging mechanisms of the auditory system

The aging of the auditory system is a multifactorial, multilayered complex physiological and pathological process, with its main mechanisms encompassing widespread changes from peripheral receptors to central neural pathways. Firstly, the structural degeneration of the peripheral auditory system is the core of age-related hearing loss (ARHL). This is primarily related to the damage and dysfunction of cochlear sensory hair cells, which are the key structures for converting sound mechanical energy into neural signals (Eshel et al., 2024; Cornejo-Sanchez et al., 2025; Li et al., 2026). A study has reported that cochlear hair cell loss is the leading cause of sensory deficit in ARHL (Wu et al., 2020). As people age, ARHL is associated with a gradual increase in systemic inflammation and a corresponding decline in hearing thresholds (Verschuur et al., 2012). This suggests that inflammation plays a key role in the progression of ARHL, and that pyroptosis may contribute to cochlear hair cell loss during aging (Yang X. et al., 2023). miR-34a has recently been found to serve as a key regulator of different types of hearing loss (Safabakhsh et al., 2022). miR-34a increases with age in several organs such as heart and blood vessels and serves a key role in age-associated functional impairment. By applying the miR-inhibitor, mitochondrial dysfunction, apoptosis and pyroptosis were reversed *in vitro* and *in vivo*, suggesting that miR-34a served as a key modulator of the aging process (Wang et al., 2025). Single-cell transcriptome analysis further reveals significant transcriptomic changes in inner and outer hair cells in elderly individuals (Eshel et al., 2024; Kaufman et al., 2025). To unveil the gene expression dynamics of cochlear aging at temporal resolution, Sun et al. constructed a high-throughput scRNA-seq atlas of the mouse cochlea spanning five time points: 1, 2, 5, 12, and 15 months of age. Their analysis pinpoints loss of proteostasis and elevated apoptosis as the hallmark features of cochlear aging, highlights unexpected age-related transcriptional fluctuations in intermediate cells localized in the stria vascularis (SV) and demonstrates that upregulation of endoplasmic reticulum (ER) chaperon protein HSP90AA1 mitigates ER stress-induced damages associated with aging (Sun et al., 2023). Meanwhile, the spiral ganglion neurons (SGNs) that connect hair cells to the central nervous system also undergo degeneration, characterized by a reduction in synaptic connections between inner hair cells, known as “synaptic degeneration,” which directly disrupts the precise transmission of sound signals to the brain (Xu et al., 2023; Zhang et al., 2024). Spiral ganglion neurons themselves also experience age-related neuroinflammation and accelerated degeneration processes (Cassinotti et al., 2022). Additionally, the stria vascularis, which maintains the ionic balance and metabolic support of the cochlear endolymph, undergoes degeneration, leading to a decrease in endolymphatic potential and thus weakening the driving force for hair cell transduction currents (Jung S. H. et al., 2024; Zheng et al., 2024). During this process, the dysfunction of potassium ion channels on hair cells such as Kv7.4 and KCNQ4 has also been confirmed to play an important role (Jung S. H. et al., 2024). Secondly, the molecular and cellular mechanisms driving these structural degenerations are intricate. Oxidative stress and chronic inflammation are considered core driving factors, among which the decline in mitochondrial function and related oxidative damage play a key role in the degeneration of hair cells and neurons (Cornejo-Sanchez et al., 2023; Pyott et al., 2024; Yeo et al., 2024). Cellular senescence, as an important cellular fate, also exists in the aging cochlea, promoting local inflammation and tissue dysfunction through pathways such as the release of senescence-associated secretory phenotype (SASP) factors, thereby accelerating the progression of ARHL (He et al., 2024). Finally, the effects of aging extend to the central auditory system. Peripheral hearing loss can trigger abnormal neural responses and frequency map reorganization in central auditory pathways (He et al., 2021). It is worth noting that traditional pure-tone hearing tests may underestimate the important role of the decline in central auditory processing ability in ARHL and its association with cognitive disorders (such as dementia) (Fatima Heredia et al., 2023; Kim et al., 2025). Studies have shown that cochlear dysfunction is related to brain atrophy and a decline in cognitive scores, highlighting the importance of high-quality peripheral (Cornejo-Sanchez et al., 2025) signal input for maintaining central function (Yang X. et al., 2023).

### Genetic factors and gene mutations

The genetic basis of age-related hearing loss (ARHL) is highly polygenic and complex, involving both common and rare genetic variants. Multiple studies confirm that rare variants in Mendelian hearing loss genes significantly contribute to ARHL pathogenesis. Exome data analysis from large population cohorts (e.g., UK Biobank) has identified several Mendelian hearing loss genes associated with ARHL, where both single-variant analysis and rare-variant aggregate association analyses demonstrate that these rare variants significantly elevate ARHL risk (Cornejo-Sanchez et al., 2023; Cornejo-Sanchez et al., 2025). Concurrently, genome-wide association studies (GWAS) have extensively established those common genetic variants, such as single nucleotide polymorphisms (SNPs), are significant risk factors for ARHL. Large-scale GWAS have discovered dozens of genetic loci associated with an increased risk of ARHL (Eshel et al., 2024; Ninoyu and Friedman, 2024; Kaufman et al., 2025; Li et al., 2026).

Advances in research methodologies continue to uncover novel susceptibility genes and key cell types. For instance, a large-scale single-cell transcriptome-wide association study (scTWAS) successfully identified new susceptibility genes and specific cell types associated with ARHL, highlighting the importance of delineating genetic contributions at single-cell resolution (Li et al., 2026). Regarding the functional mechanisms of specific genes, GWAS identified KLHDC7B, a gene of previously unknown function, which is specifically expressed in sensory hair cells within the mouse cochlea. KLHDC7B knockout models show elevated hearing thresholds, indicating its crucial role in hair cell function (Kaufman et al., 2025). Otherwise, a study has found that mutations in KCNQ4 are linked to progressive HL (DFNA2), noise-induced hearing loss, and presbycusis, leading to K^+^ accumulation, cellular stress, and OHC death (Rias et al., 2025). Although KCNQ4 mutations are primarily associated with hereditary progressive hearing loss (specifically autosomal dominant nonsyndromic hearing loss type DFNA2), rather than typical age-related hearing loss (ARHL). However, some studies suggest that KCNQ4 dysfunction may contribute to the pathological processes of ARHL, potentially through interactions with factors such as noise exposure and age-related cochlear degeneration (Rias et al., 2025). Furthermore, known hereditary deafness genes also participate in ARHL progression: haploinsufficiency of the connexin gene GJB2 accelerates ARHL development (Xu et al., 2023); mutations in the OPA1 gene, encoding the inner mitochondrial membrane fusion protein, implicate mitochondrial dysfunction as a potential mechanism underlying ARHL (Zhang et al., 2024); and the gene Cisd2, involved in maintaining redox balance, relates to resistance against oxidative stress and cellular senescence, factors associated with ARHL-related neuronal issues(Chen et al., 2024) .

Genetic research on ARHL faces challenges due to its polygenic complexity. Recent large-scale GWAS and phenome-wide association studies (PheWAS) are dedicated to dissecting its intricate genetic architecture, identifying causal variants, and understanding gene pleiotropy (Ninoyu and Friedman, 2024). Integrating GWAS results with other omics data, such as epigenetics, aids in exploring potential biological pathways and causal mechanisms (Eshel et al., 2024). Methodologies are also evolving: scTWAS has emerged as a powerful new tool for identifying susceptibility genes and specific pathogenic cell types (Li et al., 2026), while exome sequencing and rare-variant aggregate association analysis remain key methods for assessing the contribution of rare variants (Cornejo-Sanchez et al., 2025).

Beyond genetic factors themselves, their interaction with environmental factors is equally important. Research indicates that lifestyle factors such as exposure to noise may accelerate the progression of ARHL (Paciello et al., 2023a), a critical avenue for future research involves delving deeper into the combined associations among genetic factors, lifestyle/environmental factors, and adherence to healthy lifestyle practices (Jung S. H. et al., 2024).

### Influence of environmental factors and lifestyle

The onset and progression of age-related hearing loss (ARHL) are influenced not only by genetics and aging but also significantly shaped by modifiable factors, including environmental exposures and lifestyle choices (Henshaw et al., 2023). Among environmental factors, chronic or high-intensity noise exposure stands as one of the primary modifiable risk factors. It is associated with an accelerated decline in pure-tone hearing thresholds, indicating that noise can hasten the progression of ARHL (Paciello et al., 2021; Ramkumar et al., 2021; Paciello et al., 2023a). Noise-induced hearing loss (NIHL) itself is recognized as a risk factor for ARHL. Aged animals raised in quiet environments did not lose hair cells until well past the middle of the lifespan, and the loss was small, whereas human temporal bone specimens have found stable and large loss of hair cells throughout the life (Kujawa and Liberman, 2019). This suggests that noise exposure synergizes with aging in the development of presbycusis, and the two conditions may interact through shared molecular mechanisms, such as oxidative stress (Rivas-Chacon et al., 2021; Maniaci et al., 2024).

Furthermore, exposure to certain ototoxic chemicals represents a potential contributing factor to global hearing loss (Tang et al., 2023; Suzuki et al., 2024). These substances may directly damage cochlear cells or exacerbate oxidative stress.

Regarding lifestyle, several modifiable behaviors are associated with an increased risk of ARHL. Smoking can damage the cochlea by promoting inflammation and oxidative stress (Ramkumar et al., 2021; Ju et al., 2022; Meng et al., 2025). Unhealthy dietary habits, such as insufficient intake of antioxidants, are correlated with increased risk (Tang et al., 2023; Suzuki et al., 2024). Conversely, a diet rich in polyphenols like cocoa demonstrates potential protective effects due to its antioxidant and anti-inflammatory properties (Kishimoto-Urata et al., 2022; Fatima Heredia et al., 2023). Meanwhile, physical inactivity is also a risk factor, while regular exercise may reduce risk by improving overall metabolic health (Tang et al., 2023; Suzuki et al., 2024). Otherwise, chronic lifestyle diseases, including obesity and diabetes, indirectly promote the development of hearing loss by exacerbating oxidative stress and inflammation (Xu et al., 2024; Meng et al., 2025).

### The role of inflammation and oxidative stress

Recently, oxidative stress was confirmed as a core mechanism in the pathogenesis of ARHL, primarily causing damage, dysfunction, and accelerated senescence of cochlear cells through the generation of excessive reactive oxygen species (ROS). ROS attack cochlear cells, such as hair cells and spiral ganglion neurons, disrupting mitochondrial function, leading to metabolic dysregulation and cell death, ultimately causing hearing loss (Lee et al., 2025). Hydrogen peroxide (H2O2) is commonly used *in vitro* to induce cellular senescence mimicking ARHL pathology, and excessive ROS-triggered NLRP3 inflammasome activation is crucial for ARHL pathogenesis (Rivas-Chacon et al., 2021). Oxidative stress accelerates cochlear cell aging via oxidative DNA damage responses (DDRs), resulting in auditory functional decline (Hou et al., 2022; Tang et al., 2023). Additionally, various stressors, including increased free radicals, damage mitochondria, contributing to cellular malfunction, viability compromise, and ultimately functional decline, correlating with progressive hearing loss in ARHL (Zheng et al., 2024). Oxidative stress serves as a common nexus connecting ARHL with factors like noise exposure and lifestyle diseases. For instance, noise-induced hearing loss (NIHL) and ototoxic drug exposure both exacerbate ARHL through ROS generation (Paciello et al., 2023a; Chen et al., 2024). ROS play a pivotal role in the pathogenesis of NIHL, ARHL, and sudden hearing loss, suggesting potential preventive benefits of antioxidants (Yang X. et al., 2023). Concurrently, oxidative stress is closely linked to modifiable risk factors like smoking and poor diet (Chen et al., 2024). Recently, studies have indicated that interventions targeting oxidative stress are promising. Antioxidants such as curcumin and cocoa polyphenols have demonstrated protective effects in animal models by mitigating oxidative stress-induced damage (Chen et al., 2024; Maniaci et al., 2024). For example, curcumin suppresses H_2_O_2_-induced oxidative stress and improves cochlear hair cell function and hearing (Li et al., 2023).

Simultaneously, inflammation represents another key mechanism in ARHL, interacting with oxidative stress to collectively promote hearing loss. Inflammatory responses are triggered via pathways such as the NLRP3 inflammasome, releasing inflammatory cytokines such as IL-6 (Bazard et al., 2021), causing cochlear tissue damage and chronic inflammation. Inflammatory markers are significantly elevated in human and animal ARHL, associated with “inflammaging” (Bazard et al., 2021). Inflammation causes tissue damage and dysfunction, leading to cochlear cell death, synapse loss, and neurodegeneration (Paciello et al., 2021; Cassinotti et al., 2022; Yang X. et al., 2023). Inflammation plays a direct role in the development of ARHL; for instance, lipopolysaccharide (LPS)-activated inflammatory pathways can damage inner ear hair cells (Yang X. et al., 2023). In noise-induced models, inflammation participates in cochlear dysfunction and correlates with downregulation of PPAR signaling pathways (Paciello et al., 2021). Moreover, inflammation often arises as a downstream effect of oxidative stress, creating a vicious cycle that further amplifies cochlear damage (Paciello et al., 2023a). Studies indicate oxidative stress is the primary element of cochlear damage, while increased inflammation can be considered a direct consequence of ROS production (Paciello et al., 2023a). Moreover, redox status imbalance serves as a common pathological mechanism linking hearing loss and cognitive impairment, with inflammation activated in both the cochlea and the brain (Paciello et al., 2023b). Chronic inflammation is also associated with ARHL comorbidities, such as cognitive decline and frailty syndrome (Sardone et al., 2021; Fatima Heredia et al., 2023).

In ARHL pathogenesis, oxidative stress and inflammation are not isolated but highly interactive mechanisms (Tang et al., 2023). Oxidative stress often acts as the initiating event, triggering inflammatory pathways. In turn, the inflammatory response generates more ROS, establishing a positive feedback loop that accelerates cochlear cell damage and functional loss. Reducing oxidative stress can indirectly suppress inflammation, indicating oxidative stress as the dominant factor (Paciello et al., 2021). Oxidative stress and inflammation lead to cellular senescence and death together, and they synergistically promote cochlear cell senescence, mitochondrial dysfunction, and apoptosis, resulting in loss of hair cells and ganglion neurons (Zhang et al., 2021; Liu et al., 2023). Comparative studies suggest antioxidant therapy may be more effective than anti-inflammatory treatment alone, implying oxidative stress is a more fundamental target (Paciello et al., 2021).

## Treatment of presbycusis

### Conventional therapeutic approaches for age-related hearing loss

Currently, traditional treatment approaches for age-related hearing loss (ARHL) primarily revolve around hearing aids, explorations of experimental drugs, and preventive measures, though their overall efficacy remains limited. Hearing aids are the most commonly used traditional treatment for ARHL, aiming to improve hearing sensitivity by amplifying sounds (Asakawa et al., 2024). Although hearing aids are widely regarded as beneficial, only about 15% of those who could benefit from them actually use them. This low uptake is largely attributed to issues such as cost, concerns about appearance, discomfort, and skepticism regarding their advantages (Chien and Lin, 2012). Existing research indicates that the use of hearing aids is considered an effective therapeutic intervention, but key unresolved questions remain regarding their impact on cognitive function, such as whether ARHL and cognitive impairment are causally related, and whether hearing aids can effectively reduce the risk of cognitive impairment or dementia (Asakawa et al., 2024). Longitudinal cohort studies have confirmed a strong association between ARHL and cognitive function, with an increased risk of cognitive decline as the severity of hearing loss progresses, though the specific efficacy of hearing aids in this process remains unclear (Zhao et al., 2024). These findings suggest that while hearing aids are widely adopted, their long-term benefits for ARHL-related complications require further experimental or clinical evidence (Asakawa et al., 2024).

In terms of drug therapy, it is generally believed that ARHL lacks effective pharmacological treatment options. Research clearly states that due to the lack of comprehensive trials, no appropriate treatment drugs are currently available (Hu et al., 2023). Nevertheless, several studies have investigated the potential of pharmacological interventions. For instance, both antioxidants and anti-inflammatory agents have demonstrated promise in preclinical research, as reactive oxygen species are implicated in various types of hearing loss and antioxidants may aid in the prevention or treatment of related auditory disorders. However, these findings are based on accumulated experimental evidence and have not yet been translated into clinical practice (Lee et al., 2025). Given that oxidative stress is a central trigger of ARHL, research has focused on antioxidant factors such as Sestrin2 for mitigating oxidative damage, though their underlying mechanisms remain unclear (Lee et al., 2025). A recent study reported that metformin enhanced the survival of senescent auditory cells and improved mitochondrial function by decreasing the production of reactive oxygen species, while also suppressing ferroptosis *in vitro*. *In vivo*, metformin improved auditory function in C57BL/6J mice, lowered cochlear levels of iron and malondialdehyde, and extended the survival of hair cells. Additionally, both *in vitro* and *in vivo* experiments showed that metformin increased the expression of SIRT1, PINK1, and GPX4. These findings suggest that metformin may have therapeutic potential for presbycusis. Nevertheless, this finding has so far only been demonstrated in mice, and its effectiveness in humans remains unproven. Additionally, other investigational drugs, including those targeting senescent cells, have shown potential in early animal studies to delay cochlear aging and the progression of ARHL (Lee et al., 2025), while studies on curcumin have explored its effects in reducing oxidative stress (Li et al., 2023). Despite these advances, all such findings remain at the preclinical stage, and no effective therapies are currently available to prevent or slow the progression of ARHL (Cassinotti et al., 2022), underscoring the limitations of current drug-based approaches. The efficacy of rehabilitation training in ARHL management has not been confirmed or systematically discussed, and specialized rehabilitation strategies for ARHL are currently lacking (Zhao et al., 2024). Hearing protection is indirectly emphasized as a preventive measure. Noise-induced hearing loss is recognized as a risk factor for ARHL, with noise exposure potentially accelerating sensory aging and cognitive decline (Paciello et al., 2023a);

### Novel therapeutic strategies

Gene therapy has shown considerable promise in the treatment of hearing loss, primarily by correcting or replacing pathogenic genes to restore auditory function. This approach centers on the safe and efficient delivery of therapeutic genes to cochlear cells, addressing the molecular underpinnings of hearing loss (Du et al., 2023). Its efficacy has been demonstrated in animal models; for instance, in aged mice with TMPRSS3 mutations, AAV2-hTMPRS3 gene therapy successfully restored hearing to near-wild-type levels with sustained long-term effects, paving the way for potential human applications (Du et al., 2023). Additionally, defects in the GJB2 (Cx26) gene have been linked to accelerated ARHL progression, further supporting gene replacement as a viable strategy (Xu et al., 2023; Sun Q. et al., 2025). Adeno-associated virus (AAV) has become the major delivery platform for inner ear gene therapy due to its favorable safety profile and high transduction efficiency (Jang et al., 2025). In recent years, optimization of AAV vectors has significantly improved their targeting and expression efficiency in cochlear cells. For instance, self-complementary AAV (scAAV) can effectively restore CLIC5 protein expression, prevent cochlear structural degeneration, and preserve auditory and vestibular functions even at low titers when delivering the Clic5 gene to Clic5-deficient mice, suggesting that it can reduce therapeutic doses and mitigate potential toxicity (Hahn et al., 2025). In addition, researchers identified the outer hair cell-specific enhancer B8 through the *in vitro* and *in vivo* Augmented RNA-level Identification of Byclic Enhancer Regions (ARBITER) workflow and integrated it into AAV vectors, successfully restoring hearing in a Slc26a5 knockout mouse model, which highlights the importance of promoter/enhancer element optimization for cell type-specific expression (Zhao S. et al., 2025). Nonetheless, age-related hearing loss is associated with certain genes, the genetic therapies discussed above are primarily aimed at hereditary hearing loss. It remains unclear whether they have a direct effect on age-related hearing loss. Simultaneously, several challenges persist, such as improving the targeting specificity of AAV, overcoming the intricate anatomy of the cochlea, and minimizing immune reactions (Zhao S. et al., 2025; Liao et al., 2026). Emerging technologies, such as dual-vector systems and CRISPR-based gene editing, are under investigation to improve precision (Shubina-Oleinik et al., 2021; Wu et al., 2025).

Cassinotti et al. demonstrated that overexpressing neurotrophin-3 (Ntf3) in the cochleae of middle-aged mice helped prevent age-related synaptic loss in inner hair cells and slowed the progression of age-related hearing loss (Cassinotti et al., 2022). Additionally, oral administration of selegiline, a neuroprotective antiparkinsonian agent, significantly improved high-frequency hearing loss in mice with moderate impairment, but showed no benefit in those with rapidly progressing hearing loss (Szepesy et al., 2021). While drug therapy for presbycusis holds significant promise, current research is largely limited to animal studies and early-stage clinical trials. For widespread clinical use, further challenges regarding efficacy, safety, optimal delivery methods, and dosing must be addressed.

Stem cell therapy aims to regenerate or rescue damaged hair cells and neurons, offering a regenerative approach to ARHL. Mesenchymal stem cells (MSCs) have demonstrated the capacity to differentiate into inner ear cell subtypes and deliver Apelin protein via thereby ameliorating inflammation and oxidative stress in ARHL (Xu et al., 2025). Stem cell-based interventions are recognized as potential strategies for replacing or repairing hair cells that do not regenerate spontaneously (Cumpata et al., 2024; Li et al., 2025), with single-cell transcriptomic analyses further validating the therapeutic potential of MSCs in ARHL models (Xu et al., 2025). However, key obstacles persist, such as ensuring the survival of transplanted cells, achieving precise differentiation, and integrating new cells functionally with host tissues (Takeda et al., 2021; Xu et al., 2025). Moreover, existing ARHL animal models, such as those based on natural aging or D-galactose induction, have limitations including long experimental cycles, significant individual variability, and inconsistent pathological features, all of which hinder reliable evaluation of treatment efficacy. In addition, improvements in hearing function need to be validated through a combination of behavioral, electrophysiological, and histological assessments, as reliance on a single measure may lead to misinterpretation.

Nanotechnology in hearing loss treatment primarily involves designing nanoparticle carriers to enhance the targeted delivery of therapeutic agents or genes. When combined with gene therapy, nanoparticles can function as non-viral vectors, protecting genetic material, targeting specific cochlear cells, and reducing invasiveness, thus driving innovation in hearing loss therapeutics (Foster et al., 2023; Liu Y. et al., 2025). These systems can overcome barriers such as the blood-labyrinth barrier and they are widely used for localized delivery of drugs for various types of hearing loss, including ARHL. Drug-loaded nanoparticles administered via the tympanic cavity or intracochlear injection enable targeted intervention against pathological mechanisms, such as oxidative stress (Cosentino et al., 2024; Sun G. et al., 2025). Innovative delivery strategies, including tympanic cavity injections combined with nanocarriers, can enhance local efficacy and minimize systemic toxicity (Liao et al., 2026). Nanotechnology thus offers a versatile and personalized platform for ARHL treatment (Cosentino et al., 2024; Liu Y. et al., 2025). The use of nanocarriers for targeted delivery to the cochlea holds great potential for clinical translation. Although nanotechnology has shown significant promise in the treatment of hearing loss, its clinical translation and application still face several critical challenges and limitations. The transition of nanotherapies from laboratory research to clinical use is hindered by requirements for long-term biocompatibility testing, scalable manufacturing processes, individual patient differences, and complex regulatory approval procedures. These factors considerably delay the practical clinical application of nanocarrier systems. While nanoparticles can assist in crossing the blood-labyrinth barrier, efficiently and selectively targeting specific cochlear cells remains technically challenging. Current strategies—such as round window membrane penetration, magnetic navigation, and ultrasound-assisted delivery—are effective in animal models, but their safety and applicability in the complex anatomy of the human ear need further validation. Additionally, some nanomaterials may trigger local or systemic immune responses, inflammation, or unknown long-term toxicity. In the treatment of chronic hearing loss requiring repeated administration, such as ARHL, the cumulative effects and potential disruption of the cochlear microenvironment by nanocarriers require systematic assessment. Furthermore, there is currently a lack of long-term efficacy and safety data specific to the human cochlear microenvironment.

### Advances in early diagnosis and early intervention

Novel intervention strategies for age-related hearing loss (ARHL) now extend beyond post-onset treatment to encompass early screening, preventive lifestyle modifications, and precision management based on emerging biomarkers. Together, these approaches form a multidimensional framework for ARHL prevention and control.

Early detection is a prerequisite for effective intervention. Integrating auditory tasks with neuroimaging techniques has significantly improved the early diagnosis of ARHL and its associated cognitive decline. For example, dual auditory oddball and cognitive task paradigms, combined with functional connectivity analyses, can assess auditory processing and cognitive function in ARHL patients (Zhao et al., 2024; Liu Y. et al., 2025). Application of these paradigms yields dynamic brain connectivity data, revealing deficits such as impaired spatial selective attention, and thereby facilitates early diagnosis. These methods help identify the early association between ARHL and cognitive decline, providing objective evidence for timely intervention.

A substantial body of evidence indicates that lifestyle interventions targeting modifiable risk factors are effective in preventing and delaying ARHL. These interventions primarily exert their effects through antioxidant and anti-inflammatory mechanisms. Dietary components rich in polyphenols, such as cocoa, have attracted attention for their potent anti-inflammatory and antioxidant properties, potentially slowing ARHL progression by alleviating oxidative stress (Del Mar Rivas-Chacon et al., 2022; Fatima Heredia et al., 2023). Importantly, comprehensive health behavior scores from large cohort studies show that adherence to a healthy lifestyle—including non-smoking, high physical activity, and a high-quality diet—markedly reduces ARHL risk (Yevenes-Briones et al., 2022; Tang et al., 2023). This protective effect is especially pronounced in individuals with a high genetic risk, where healthy behaviors substantially lower the likelihood of ARHL (Jung S. H. et al., 2024). Specifically, nonsmoking and high physical activity are directly associated with reduced risk, while intake of antioxidant-rich foods helps mitigate oxidative stress-induced cochlear damage (Yevenes-Briones et al., 2022; Tang et al., 2023; Jung S. H. et al., 2024).

The discovery of novel biomarkers offers new opportunities for early risk prediction and targeted intervention in ARHL. These biomarkers are mainly linked to key pathological mechanisms such as inflammation, oxidative stress, and genetic susceptibility. Inflammatory markers like interleukin-6 and leukocyte count have shown significant associations with ARHL in both human and animal studies, highlighting the critical role of “inflammaging” in disease development (Bazard et al., 2021; Zheng et al., 2024). Changes in oxidative stress-induced DNA damage responses and antioxidant factors such as Sestrin2 may serve as potential biomarkers for monitoring disease progression (Bazard et al., 2021; Suzuki et al., 2024; Zheng et al., 2024). Additionally, genome-wide association studies and single-cell transcriptomic analyses have identified genetic variants—such as those in KLHDC7B—associated with cochlear hair cell function (Kaufman et al., 2025; Li et al., 2026). The identification of senescence-related biomarkers not only deepens our understanding of ARHL pathogenesis but also provides therapeutic targets for emerging treatments such as senolytics (Liu et al., 2023; Eshel et al., 2024; Jung J. et al., 2024; Chen et al., 2025). These biomarkers facilitate the early identification of high-risk individuals and guide the development of precision prevention and treatment strategies.

## Conclusion and perspectives

Age-related hearing loss (ARHL) is a complex disorder involving multiple factors and mechanisms. Its high prevalence and profound social and health impacts have made it a major global public health concern. This review highlights that the pathophysiology of ARHL encompasses peripheral auditory degeneration, central roles of oxidative stress and inflammation, genetic susceptibility, and the interplay of environmental factors. Although conventional interventions such as hearing aids can partially alleviate symptoms, there are currently no effective therapies to halt or reverse disease progression.

In recent years, advances in molecular biology, gene editing, nanotechnology, and regenerative medicine have shifted ARHL treatment strategies from symptomatic management toward etiological therapies. Gene therapy has achieved success in correcting specific genetic defects in animal models; stem cell therapy offers hope for the regeneration of hair cells and neurons; nanoparticle delivery systems have the potential to overcome the blood-cochlear barrier for precise drug administration; and the identification of early biomarkers and lifestyle interventions provides feasible avenues for prevention and disease delay. While most of these emerging strategies remain in preclinical or early clinical stages, they demonstrate significant translational potential.

Through the close integration of basic research and clinical practice, effective prevention and treatment of ARHL may be achieved in the future, ultimately improving the quality of life and health outcomes for the aging population.

## References

[B1] Affortit C. Blanc F. Nasr J. Ceccato J. C. Markossian S. Guyot R. et al. (2022). A disease-associated mutation in thyroid hormone receptor alpha1 causes hearing loss and sensory hair cell patterning defects in mice. *Sci. Signal.* 15:eabj4583. 10.1126/scisignal.abj4583 35700264

[B2] Affortit C. Casas F. Ladrech S. Ceccato J. C. Bourien J. Coyat C. et al. (2021). Exacerbated age-related hearing loss in mice lacking the p43 mitochondrial T3 receptor. *BMC Biol.* 19:18. 10.1186/s12915-021-00953-1 33526032 PMC7852282

[B3] Ahmed S. Vaden K. I. Lewis M. A. Leitao D. Steel K. P. Dubno J. R. et al. (2025). Large-scale audiometric phenotyping identifies distinct genes and pathways involved in hearing loss subtypes. *Commun. Biol.* 8:1515. 10.1038/s42003-025-08778-2 41174146 PMC12579245

[B4] Asakawa T. Yang Y. Xiao Z. Shi Y. Qin W. Hong Z. et al. (2024). Stumbling blocks in the investigation of the relationship between age-related hearing loss and cognitive impairment. *Perspect. Psychol. Sci.* 19 137–150. 10.1177/17456916231178554 37410696

[B5] Bai X. Liao B. Hu W. H. Wang M. Q. Sun Y. Chen X. B. et al. (2025). Cochlear endoplasmic reticulum stress causes connexin 26 degradation is involved in age-related hearing loss. *iScience* 28:113891. 10.1016/j.isci.2025.113891 41321625 PMC12663731

[B6] Bazard P. Pineros J. Frisina R. D. Bauer M. A. Acosta A. A. Paganella L. R. et al. (2021). Cochlear inflammaging in relation to ion channels and mitochondrial functions. *Cells* 10:2761. 10.3390/cells10102761 34685743 PMC8534887

[B7] Boons J. H. C. Dingemanse G. Vinke E. J. Kremer B. Vernooij M. W. Goedegebure A. (2025). Loss of microstructural integrity in left hemispheric white matter tracts is associated with poorer digits in noise understanding. *Geroscience* 48 1063–1077. 10.1007/s11357-025-01707-5 40418507 PMC12972419

[B8] Bowl M. R. Dawson S. J. (2019). Age-related hearing loss. *Cold Spring Harb. Perspect. Med.* 9:a033217. 10.1101/cshperspect.a033217 30291149 PMC6671929

[B9] Brewster K. K. Golub J. S. Rutherford B. R. (2021). Neural circuits and behavioral pathways linking hearing loss to affective dysregulation in older adults. *Nat. Aging* 1 422–429. 10.1038/s43587-021-00065-z 37118018 PMC10154034

[B10] Cassinotti L. R. Ji L. Borges B. C. Cass N. D. Desai A. S. Kohrman D. C. et al. (2022). Cochlear Neurotrophin-3 overexpression at mid-life prevents age-related inner hair cell synaptopathy and slows age-related hearing loss. *Aging Cell* 21:e13708. 10.1111/acel.13708 36088647 PMC9577954

[B11] Castelli V. d’Angelo M. Zazzeroni F. Vecchiotti D. Alesse E. Capece D. et al. (2023). Intranasal delivery of NGF rescues hearing impairment in aged SAMP8 mice. *Cell Death Dis.* 14:605. 10.1038/s41419-023-06100-8 37704645 PMC10499813

[B12] Chen H. K. Wang Y. H. Lei C. S. Guo Y. R. Tang M. C. Tsai T. F. et al. (2024). Loss of Cisd2 Exacerbates the Progression of Age-Related Hearing Loss. *Aging Dis.* 16 2468–2482. 10.14336/ad.2024.1036 39226169 PMC12221414

[B13] Chen Y. Huang H. Luo Y. Wu H. Deng W. Min X. et al. (2025). Senolytic treatment alleviates cochlear senescence and delays age-related hearing loss in C57BL/6J mice. *Phytomedicine* 142:156772. 10.1016/j.phymed.2025.156772 40253743

[B14] Chien W. Lin F. R. (2012). Prevalence of hearing aid use among older adults in the United States. *Arch. Intern. Med.* 172 292–293. 10.1001/archinternmed.2011.1408 22332170 PMC3564585

[B15] Clark D. P. Q. Zhou Z. Hussain S. M. Tran C. Britt C. Storey E. et al. (2024). Low-dose aspirin and progression of age-related hearing loss: A Secondary analysis of the ASPREE randomized clinical trial. *JAMA Netw. Open* 7:e2424373. 10.1001/jamanetworkopen.2024.24373 39052288 PMC11273233

[B16] Cornejo-Sanchez D. M. Bharadwaj T. Dong R. Wang G. T. Schrauwen I. DeWan A. T. et al. (2025). Mendelian non-syndromic and syndromic hearing loss genes contribute to presbycusis. *Eur. J. Hum. Genet.* 33 758–767. 10.1038/s41431-025-01789-x 40055553 PMC12185688

[B17] Cornejo-Sanchez D. M. Li G. Fabiha T. Wang R. Acharya A. Everard J. L. et al. (2023). Rare-variant association analysis reveals known and new age-related hearing loss genes. *Eur. J. Hum. Genet.* 31 638–647. 10.1038/s41431-023-01302-2 36788145 PMC10250305

[B18] Cosentino A. Agafonova A. Modafferi S. Trovato Salinaro A. Scuto M. Maiolino L. et al. (2024). Blood-labyrinth barrier in health and diseases: Effect of hormetic nutrients. *Antioxid. Redox Signal.* 40 542–563. 10.1089/ars.2023.0251 37565276

[B19] Cumpata A. J. Labusca L. Radulescu L. M. (2024). Stem cell-based therapies for auditory hair cell regeneration in the treatment of hearing loss. *Tissue Eng. Part. B Rev.* 30 15–28. 10.1089/ten.TEB.2023.0084 37440318

[B20] Del Mar Rivas-Chacon L. Yanes-Diaz J. de Lucas B. Riestra-Ayora J. I. Madrid-Garcia R. et al. (2022). Preventive effect of cocoa flavonoids via suppression of oxidative stress-induced apoptosis in auditory senescent cells. *Antioxidants* 11:1450. 10.3390/antiox11081450 35892652 PMC9330887

[B21] Ding R. Huang W. Shen C. Pan Y. Zhong Y. Kong B. et al. (2025). DLK/JNK3 upregulation aggravates hair cell senescence in mice cochleae via excessive autophagy. *Aging Cell.* 24:e70099. 10.1111/acel.70099 40356329 PMC12341777

[B22] Du W. Ergin V. Loeb C. Huang M. Silver S. Armstrong A. M. et al. (2023). Rescue of auditory function by a single administration of AAV-TMPRSS3 gene therapy in aged mice of human recessive deafness DFNB8. *Mol. Ther.* 31 2796–2810. 10.1016/j.ymthe.2023.05.005 37244253 PMC10491991

[B23] Eshel M. Milon B. Hertzano R. Elkon R. (2024). The cells of the sensory epithelium, and not the stria vascularis, are the main cochlear cells related to the genetic pathogenesis of age-related hearing loss. *Am. J. Hum. Genet.* 111 614–617. 10.1016/j.ajhg.2024.01.008 38330941 PMC10940011

[B24] Fatima Heredia R. Riestra-Ayora J. I. Yanes-Diaz J. Thuissard Vasallo I. J. Andreu-Vazquez C. Sanz-Fernandez R. et al. (2023). Cocoa polyphenols prevent age-related hearing loss and frailty in an in vivo model. *Antioxidants* 12:1994. 10.3390/antiox12111994 38001847 PMC10669688

[B25] Foster T. Lewkowicz M. Quintas C. Ionescu C. M. Jones M. Wagle S. R. et al. (2023). Novel nanoencapsulation technology and its potential role in bile acid-based targeted gene delivery to the inner ear. *Small* 19:e2204986. 10.1002/smll.202204986 36538754

[B26] Hahn R. Taiber S. Shubina-Oleinik O. Geleoc G. S. G. Holt J. R. Avraham K. B. (2025). AAV gene therapy rescues hearing and balance in a model of CLIC5 deafness. *EMBO Mol. Med.* 17 2233–2257. 10.1038/s44321-025-00275-7 40859056 PMC12423326

[B27] He J. Cabrera-Mendoza B. De Angelis F. Pathak G. A. Koller D. Curhan S. G. et al. (2024). Sex differences in the pleiotropy of hearing difficulty with imaging-derived phenotypes: A brain-wide investigation. *Brain* 147 3395–3408. 10.1093/brain/awae077 38454550 PMC11449129

[B28] He Z. H. Li M. Fang Q. J. Liao F. L. Zou S. Y. Wu X. et al. (2021). FOXG1 promotes aging inner ear hair cell survival through activation of the autophagy pathway. *Autophagy* 17 4341–4362. 10.1080/15548627.2021.1916194 34006186 PMC8726647

[B29] Henshaw H. Calvert S. Heffernan E. Broome E. E. Burgon C. Dening T. et al. (2023). New horizons in hearing conditions. *Age Ageing* 52:afad150. 10.1093/ageing/afad150 37604677 PMC10442518

[B30] Hou S. Chen P. He J. Chen J. Zhang J. Mammano F. et al. (2022). Dietary intake of deuterium oxide decreases cochlear metabolism and oxidative stress levels in a mouse model of age-related hearing loss. *Redox Biol.* 57:102472. 10.1016/j.redox.2022.102472 36162258 PMC9513171

[B31] Hu S. Sun Q. Xu F. Jiang N. Gao J. (2023). Age-related hearing loss and its potential drug candidates: A systematic review. *Chin. Med.* 18:121. 10.1186/s13020-023-00825-6 37730634 PMC10512576

[B32] Huang H. M. Chen G. S. Liu Z. Y. Meng Q. L. Li J. H. Dong H. W. et al. (2023). Age-related hearing loss accelerates the decline in fast speech comprehension and the decompensation of cortical network connections. *Neural Regen. Res.* 18 1968–1975. 10.4103/1673-5374.361530 36926721 PMC10233765

[B33] Jang S. H. Song H. G. Jung J. Gee H. Y. (2025). Recent preclinical and clinical advances in gene therapy for hereditary hearing loss. *Mol. Cells* 48:100285. 10.1016/j.mocell.2025.100285 41086995 PMC12605010

[B34] Ju M. J. Park S. K. Kim S. Y. Choi Y. H. (2022). Long-term exposure to ambient air pollutants and hearing loss in Korean adults. *Sci. Total Environ.* 820:153124. 10.1016/j.scitotenv.2022.153124 35051467

[B35] Jung J. Lee J. Kang H. Park K. Kim Y. S. Ha J. et al. (2024). miR-409-3p regulates IFNG and p16 signaling in the human blood of aging-related hearing loss. *Cells* 13:1595. 10.3390/cells13181595 39329776 PMC11429563

[B36] Jung S. H. Lee Y. C. Shivakumar M. Kim J. Yun J. S. Park W. Y. et al. (2024). Association between genetic risk and adherence to healthy lifestyle for developing age-related hearing loss. *BMC Med.* 22:141. 10.1186/s12916-024-03364-5 38532472 PMC10964689

[B37] Kaufman A. M. Silver B. Donnianni R. A. Aguilar C. Niu L. Johnson D. et al. (2025). Cross-species validation of a human age-related hearing loss candidate KLHDC7B as essential for mammalian hearing. *Commun. Biol*. 9:84. 10.1038/s42003-025-09349-1 41407909 PMC12820229

[B38] Kim J. A. Jang S. H. Joo S. Y. Kim S. J. Choi J. Y. Jung J. et al. (2025). Systematic genetic assessment of hearing loss using whole-genome sequencing identifies pathogenic variants. *Exp. Mol. Med.* 57 775–787. 10.1038/s12276-025-01428-x 40164689 PMC12046045

[B39] Kishimoto-Urata M. Urata S. Fujimoto C. Yamasoba T. (2022). Role of oxidative stress and antioxidants in acquired inner ear disorders. *Antioxidants* 11:1469. 10.3390/antiox11081469 36009187 PMC9405327

[B40] Kolo F. B. Lu S. Beiser A. S. Francis L. van Lent D. M. Goss M. et al. (2025). Association of hearing loss to brain structure and cognition in the Framingham Heart Study. *Alzheimer’s Dement.* 21:e101741. 10.1002/alz70860_101741

[B41] Krist A. H. Davidson K. W. Mangione C. M. Cabana M. Caughey A. B. Davis E. M. et al. (2021). Screening for hearing loss in older adults: US preventive services task force recommendation statement. *JAMA* 325 1196–1201. 10.1001/jama.2021.2566 33755083

[B42] Kujawa S. G. Liberman M. C. (2019). Translating animal models to human therapeutics in noise-induced and age-related hearing loss. *Hear. Res.* 377 44–52. 10.1016/j.heares.2019.03.003 30903954 PMC6534965

[B43] Lasisi A. O. Fehintola F. A. Yusuf O. B. (2010). Age-related hearing loss, vitamin B12, and folate in the elderly. *Otolaryngol. Head Neck Surg.* 143 826–830. 10.1016/j.otohns.2010.08.031 21109085

[B44] Lee J. M. Cha Y. J. Oh Y. J. Kim H. O. Kim S. S. Kim Y. J. et al. (2025). Expression and roles of free radicals and reactive oxygen species in hearing loss. *Antioxidants* 14:1397. 10.3390/antiox14121397 41462597 PMC12729874

[B45] Li N. Yan X. Huang W. Chu M. Dong Y. Song H. et al. (2023). Curcumin protects against the age-related hearing loss by attenuating apoptosis and senescence via activating Nrf2 signaling in cochlear hair cells. *Biochem. Pharmacol.* 212:115575. 10.1016/j.bcp.2023.115575 37334787

[B46] Li Y. Zeng T. Song W. Wang Y. Ren L. Yang C. et al. (2026). A single-cell transcriptome-wide association study reveals susceptibility genes for age-related hearing loss. *Genomics Proteomics Bioinformatics* Online ahead of print. 10.1093/gpbjnl/qzaf137 41495531

[B47] Li Z. Zhang Y. Xiao Z. Qi J. (2025). The reconstruction of peripheral auditory circuit: Recent advances and future challenges. *Adv. Sci.* 12:e2410494. 10.1002/advs.202410494 40125727 PMC12362778

[B48] Liao M. Zhu Q. Cheng H. Qiu Y. Xu S. Bu C. et al. (2026). Magnetic mesoporous nanoparticles loaded with lycium barbarum glycopeptide for targeted therapy of noise-triggered auditory dysfunction. *Adv. Mater*. 38:e09502. 10.1002/adma.202509502 41532206

[B49] Liu H. Bai Y. Guo M. Zhao R. Zhu J. Ni G. (2025). Dynamic brain functional connectivity in age-related hearing loss during auditory selective spatial attention. *Geroscience* Online ahead of print. 10.1007/s11357-025-02069-8 41460615 PMC13638867

[B50] Liu W. Johansson A. Rask-Andersen H. Rask-Andersen M. (2021). A combined genome-wide association and molecular study of age-related hearing loss in H. sapiens. *BMC Med.* 19:302. 10.1186/s12916-021-02169-0 34847940 PMC8638543

[B51] Liu Y. Li L. Huang P. Zha D. Deng H. (2025). Nanotechnology-enhanced gene therapy for hearing loss. *Nanoscale Horiz.* 10 2641–2667. 10.1039/d5nh00520e 40788057

[B52] Liu Y. Zhang H. Fan C. Liu F. Li S. Li J. et al. (2023). Potential role of Bcl2 in lipid metabolism and synaptic dysfunction of age-related hearing loss. *Neurobiol. Dis.* 187:106320. 10.1016/j.nbd.2023.106320 37813166

[B53] Maidment D. W. Wallhagen M. I. Dowd K. Mick P. Piker E. Spankovich C. et al. (2023). New horizons in holistic, person-centred health promotion for hearing healthcare. *Age Ageing* 52:afad020. 10.1093/ageing/afad020 36821645 PMC9949576

[B54] Maniaci A. La Via L. Lechien J. R. Sangiorgio G. Iannella G. Magliulo G. et al. (2024). Hearing loss and oxidative stress: A comprehensive review. *Antioxidants* 13:842. 10.3390/antiox13070842 39061910 PMC11274311

[B55] Medel V. Delano P. H. Belkhiria C. Leiva A. De Gatica C. Vidal V. et al. (2024). Cochlear dysfunction as an early biomarker of cognitive decline in normal hearing and mild hearing loss. *Alzheimers Dement.* 16:e12467. 10.1002/dad2.12467 38312514 PMC10835081

[B56] Meng L. Liu S. Luo J. Tu Y. Li T. Li P. et al. (2025). Oxidative stress and reactive oxygen species in otorhinolaryngological diseases: Insights from pathophysiology to targeted antioxidant therapies. *Redox Rep.* 30:2458942. 10.1080/13510002.2025.2458942 39894944 PMC11792148

[B57] Nieman C. L. (2025). Practical approaches to identifying & addressing hearing loss among persons living with dementia. *Alzheimer’s Dement.* 21:101399. 10.1002/alz70857_101399

[B58] Ninoyu Y. Friedman R. A. (2024). The genetic landscape of age-related hearing loss. *Trends Genet.* 40 228–237. 10.1016/j.tig.2023.12.001 38161109

[B59] Ohlemiller K. K. (2004). Age-related hearing loss: The status of Schuknecht’s typology. *Curr. Opin. Otolaryngol. Head Neck Surg.* 12 439–443. 10.1097/01.moo.0000134450.99615.22 15377958

[B60] Paciello F. Pisani A. Rinaudo M. Cocco S. Paludetti G. Fetoni A. R. et al. (2023a). Noise-induced auditory damage affects hippocampus causing memory deficits in a model of early age-related hearing loss. *Neurobiol. Dis.* 178:106024. 10.1016/j.nbd.2023.106024 36724860

[B61] Paciello F. Pisani A. Rolesi R. Escarrat V. Galli J. Paludetti G. et al. (2021). Noise-induced cochlear damage involves PPAR down-regulation through the interplay between oxidative stress and inflammation. *Antioxidants* 10:1188. 10.3390/antiox10081188 34439436 PMC8388985

[B62] Paciello F. Ripoli C. Fetoni A. R. Grassi C. (2023b). Redox imbalance as a common pathogenic factor linking hearing loss and cognitive decline. *Antioxidants* 12:332. 10.3390/antiox12020332 36829891 PMC9952092

[B63] Peixoto Pinheiro B. Muller M. Bos M. Guezguez J. Burnet M. Tornincasa M. et al. (2022). A potassium channel agonist protects hearing function and promotes outer hair cell survival in a mouse model for age-related hearing loss. *Cell. Death Dis.* 13:595. 10.1038/s41419-022-04915-5 35817766 PMC9273644

[B64] Pyott S. J. Pavlinkova G. Yamoah E. N. Fritzsch B. (2024). Harmony in the molecular orchestra of hearing: Developmental mechanisms from the ear to the brain. *Annu. Rev. Neurosci.* 47 1–20. 10.1146/annurev-neuro-081423-093942 38360566 PMC11787624

[B65] Ramkumar V. Mukherjea D. Dhukhwa A. Rybak L. P. (2021). Oxidative stress and inflammation caused by cisplatin ototoxicity. *Antioxidants* 10:1919. 10.3390/antiox10121919 34943021 PMC8750101

[B66] Reed N. S. Jiang K. Deal J. A. (2025). Hearing loss among older adults: Epidemiology, disparities, and gaps in research. *Annu. Rev. Public Health* 47 41–58. 10.1146/annurev-publhealth-081524-110330 41160749 PMC12697576

[B67] Rias E. Carignano C. Castagna V. C. Dionisio L. Ballestero J. A. Paolillo G. et al. (2025). Insights into early cochlear damage induced by potassium channel deficiency. *Biochim. Biophys. Acta Mol. Cell. Res.* 1872:120030. 10.1016/j.bbamcr.2025.120030 40752593

[B68] Rivas-Chacon L. D. M. Martinez-Rodriguez S. Madrid-Garcia R. Yanes-Diaz J. Riestra-Ayora J. I. Sanz-Fernandez R. et al. (2021). Role of oxidative stress in the senescence pattern of auditory cells in age-related hearing loss. *Antioxidants* 10:1497. 10.3390/antiox10091497 34573129 PMC8464759

[B69] Ruttiger L. Sausbier M. Zimmermann U. Winter H. Braig C. Engel J. et al. (2004). Deletion of the Ca2+-activated potassium (BK) alpha-subunit but not the BKbeta1-subunit leads to progressive hearing loss. *Proc. Natl. Acad. Sci. U S A.* 101 12922–12927. 10.1073/pnas.0402660101 15328414 PMC516466

[B70] Safabakhsh S. Wijesinghe P. Nunez M. Nunez D. A. (2022). The role of hypoxia-associated miRNAs in acquired sensorineural hearing loss. *Front. Cell. Neurosci.* 16:916696. 10.3389/fncel.2022.916696 35990888 PMC9389718

[B71] Sardone R. Castellana F. Bortone I. Lampignano L. Zupo R. Lozupone M. et al. (2021). Association between central and peripheral age-related hearing loss and different frailty phenotypes in an older population in southern italy. *JAMA Otolaryngol. Head Neck Surg.* 147 561–571. 10.1001/jamaoto.2020.5334 33570584 PMC7879383

[B72] Schubert N. M. A. Reijntjes D. O. J. van Tuinen M. Vijayakumar S. Jones T. A. Jones S. M. et al. (2024). Pathophysiological processes underlying hidden hearing loss revealed in Kcnt1/2 double knockout mice. *Aging Cell* 23:e14243. 10.1111/acel.14243 39049179 PMC11488318

[B73] Sharma S. Wadkar A. Kommajosyula S. P. (2025). Muscarinic receptors at the auditory thalamocortical circuits and relevance to hearing. *Life Sci.* 369:123522. 10.1016/j.lfs.2025.123522 40044029

[B74] Shubina-Oleinik O. Nist-Lund C. French C. Rockowitz S. Shearer A. E. Holt J. R. (2021). Dual-vector gene therapy restores cochlear amplification and auditory sensitivity in a mouse model of DFNB16 hearing loss. *Sci. Adv.* 7:eabi7629. 10.1126/sciadv.abi7629 34910522 PMC8673757

[B75] Sun G. Fu X. Zheng Y. Hong G. Liu Z. Luo B. et al. (2025). Single-cell profiling identifies hair cell SLC35F1 deficiency as a signature of primate cochlear aging. *Nat. Aging* 5 1862–1879. 10.1038/s43587-025-00896-0 40542214

[B76] Sun G. Zheng Y. Fu X. Zhang W. Ren J. Ma S. et al. (2023). Single-cell transcriptomic atlas of mouse cochlear aging. *Protein Cell.* 14 180–201. 10.1093/procel/pwac058 36933008 PMC10098046

[B77] Sun Q. Tan F. Zhang L. Lu Y. Wei H. Li N. et al. (2025). Combined AAV-mediated specific Gjb2 expression restores hearing in DFNB1 mouse models. *Mol. Ther.* 33 3006–3021. 10.1016/j.ymthe.2025.03.029 40121530 PMC12266026

[B78] Suzuki Y. Hayashi K. Goto F. Nomura Y. Fujimoto C. Makishima M. (2024). Premature senescence is regulated by crosstalk among TFEB, the autophagy lysosomal pathway and ROS derived from damaged mitochondria in NaAsO(2)-exposed auditory cells. *Cell Death Discov.* 10:382. 10.1038/s41420-024-02139-4 39191766 PMC11350138

[B79] Szepesy J. Humli V. Farkas J. Miklya I. Timar J. Tabi T. et al. (2021). Chronic oral selegiline treatment mitigates age-related hearing loss in BALB/c mice. *Int. J. Mol. Sci.* 22:2853. 10.3390/ijms22062853 33799684 PMC7999597

[B80] Takeda H. Dondzillo A. Randall J. A. Gubbels S. P. (2021). Selective ablation of cochlear hair cells promotes engraftment of human embryonic stem cell-derived progenitors in the mouse organ of Corti. *Stem. Cell. Res. Ther.* 12:352. 10.1186/s13287-021-02403-9 34147129 PMC8214253

[B81] Tang D. Tran Y. Dawes P. Gopinath B. (2023). A narrative review of lifestyle risk factors and the role of oxidative stress in age-related hearing loss. *Antioxidants* 12:878. 10.3390/antiox12040878 37107253 PMC10135296

[B82] Verschuur C. A. Dowell A. Syddall H. E. Ntani G. Simmonds S. J. Baylis D. et al. (2012). Markers of inflammatory status are associated with hearing threshold in older people: Findings from the Hertfordshire Ageing Study. *Age Ageing* 41 92–97. 10.1093/ageing/afr140 22086966

[B83] Wang J. He F. Shepherd D. A. Li S. Lange K. Sung V. et al. (2024). Polygenic risk scores and hearing loss phenotypes in children. *JAMA Otolaryngol. Head Neck. Surg.* 151 56–64. 10.1001/jamaoto.2024.3659 39509092 PMC11544553

[B84] Wang Y. Yang M. Wang G. Liu W. Deng B. Yang X. et al. (2025). miR-34a induces apoptosis and pyroptosis in D-Galactose-induced aging cochlear hair cells via inhibiting TFAM and promoting mitochondrial dysfunction in vitro and in vivo. *Int. J. Mol. Med.* 56:100. 10.3892/ijmm.2025.5541 40314090 PMC12081035

[B85] Wu F. He W. Xiao Y. Wu Q. Zhang Q. Zhang T. et al. (2025). Exploring AAV-mediated gene therapy for inner ear diseases: From preclinical success to clinical potential. *Adv. Sci.* 12:e08397. 10.1002/advs.202408397 40538303 PMC12412547

[B86] Wu P. Z. O’Malley J. T. de Gruttola V. Liberman M. C. (2020). Age-related hearing loss is dominated by damage to inner ear sensory cells, not the cellular battery that powers them. *J. Neurosci.* 40 6357–6366. 10.1523/JNEUROSCI.0937-20.2020 32690619 PMC7424870

[B87] Xu K. Chen S. Bai X. Xie L. Qiu Y. Liu X. Z. et al. (2023). Degradation of cochlear Connexin26 accelerate the development of age-related hearing loss. *Aging Cell.* 22:e13973. 10.1111/acel.13973 37681746 PMC10652327

[B88] Xu M. Zhong S. Zhu N. Wang S. Wang J. Li X. et al. (2024). Oxidative and endoplasmic reticulum stress in diabetes-related hearing loss: Protective effects of thioredoxin. *Life Sci.* 359:123223. 10.1016/j.lfs.2024.123223 39515416

[B89] Xu S. Liu D. Zhang F. Tian Y. (2025). Innovative treatment of age-related hearing loss using MSCs and EVs with Apelin. *Cell. Biol. Toxicol.* 41:31. 10.1007/s10565-025-09988-4 39820591 PMC11739245

[B90] Yang J. Shao Z. Zhang D. Wang K. (2023). Pathogenesis and treatment progress in age-related hearing loss: A literature review. *Int. J. Clin. Exp. Pathol.* 16 315–320. 38059171 PMC10695747

[B91] Yang X. Wu Y. Zhang M. Zhang L. Zhao T. Qian W. et al. (2023). Piceatannol protects against age-related hearing loss by inhibiting cellular pyroptosis and inflammation through regulated Caspase11-GSDMD pathway. *Biomed. Pharmacother.* 163:114704. 10.1016/j.biopha.2023.114704 37100013

[B92] Yeo X. Y. Kwon S. Rinai K. R. Lee S. Jung S. Park R. (2024). A consolidated understanding of the contribution of redox dysregulation in the development of hearing impairment. *Antioxidants* 13:598. 10.3390/antiox13050598 38790703 PMC11118506

[B93] Yevenes-Briones H. Caballero F. F. Banegas J. R. Rodriguez-Artalejo F. Lopez-Garcia E. (2022). Association of lifestyle behaviors with hearing loss: The UK Biobank cohort study. *Mayo Clin. Proc.* 97 2040–2049. 10.1016/j.mayocp.2022.03.029 35710463

[B94] Yu M. Li J. Zeng X. (2025). Mechanistic insights into cisplatin-induced ototoxicity: The central role of transient receptor potential channels. *Front. Mol. Biosci.* 12:1694131. 10.3389/fmolb.2025.1694131 41169615 PMC12568418

[B95] Zhang A. Pan Y. Wang H. Ding R. Zou T. Guo D. et al. (2024). Excessive processing and acetylation of OPA1 aggravate age-related hearing loss via the dysregulation of mitochondrial dynamics. *Aging Cell.* 23:e14091. 10.1111/acel.14091 38267829 PMC11019136

[B96] Zhang Y. Lv Z. Liu Y. Cao H. Yang J. Wang B. (2021). PIN1 Protects hair cells and auditory HEI-OC1 cells against senescence by inhibiting the PI3K/Akt/mTOR pathway. *Oxid. Med. Cell. Longev.* 2021:9980444. 10.1155/2021/9980444 34285767 PMC8273041

[B97] Zhao R. Yue T. Xu Z. Zhang Y. Wu Y. Bai Y. et al. (2024). Electroencephalogram-based objective assessment of cognitive function level associated with age-related hearing loss. *Geroscience* 46 431–446. 10.1007/s11357-023-00847-w 37273160 PMC10828275

[B98] Zhao S. Yang Q. Yu Z. Chu C. Dai S. Li H. et al. (2025). Deciphering enhancers of hearing loss genes for efficient and targeted gene therapy of hereditary deafness. *Neuron* 113 1579–1596.e1575. 10.1016/j.neuron.2025.03.023 40262614

[B99] Zhao X. Shen T. Cao S. Liu Z. Pang W. Li M. et al. (2025). Presbycusis: Pathology, signal pathways, and therapeutic strategy. *Adv. Sci.* 12:e2410413. 10.1002/advs.202410413 40349177 PMC12362752

[B100] Zheng L. Li M. Li Y. Wu L. Naveena K. Zheng M. et al. (2024). Sestrin2 plays a protective role in age-related hearing loss by inhibiting NLRP3-inflammasome activity. *Mech. Ageing Dev.* 221:111964. 10.1016/j.mad.2024.111964 39019118

